# Immune system response during viral Infections: Immunomodulators, cytokine storm (CS) and Immunotherapeutics in COVID-19

**DOI:** 10.1016/j.jsps.2020.12.018

**Published:** 2021-01-07

**Authors:** Faheem Hyder Pottoo, Tareq Abu-Izneid, Abdallah Mohammad Ibrahim, Md. Noushad Javed, Noora AlHajri, Amar M. Hamrouni

**Affiliations:** a Department of Pharmacology, College of Clinical Pharmacy, Imam Abdulrahman Bin Faisal University, P. O. Box 1982, Dammam 31441, Saudi Arabia; b Pharmaceutical Sciences, College of Pharmacy, Al Ain University, Al Ain, Abu Dhabi, United Arab Emirates; c Fundamentals of Nursing Department, College of Nursing, Imam Abdulrahman Bin Faisal University, P.O.BOX 1982, Dammam 31441, Saudi Arabia; d Department of Pharmaceutics, School of Pharmaceutical Education and Research (SPER), Jamia Hamdard, New-Delhi, India; e Department of Epidemiology and Population Health, College of Medicine, Khalifa University, United Arab Emirates

**Keywords:** Acute respiratory distress syndrome (ARDS), SARS-CoV, MERS-CoV, SARS-CoV-2, COVID-19, Cytokine storm, Immunomodulators, Immunotherapeutics

## Abstract

Coronaviruses are non-segmented and single stranded positive-sense RNA (+ssRNA) viruses. To date, 06 human coronaviruses (HCoVs) are reported; α-CoVs (HCoVs-NL63 and HCoVs-229E) and β-CoVs (HCoVs-OC43, HCoVs-HKU1, SARS-CoV, MERS-CoV). While, novel coronavirus (SARS-CoV-2) is the most recent member. The genome sequence of SARS-CoV-2 is 82% similar to SARS–COV-1. The compelling evidences link the progression of viral infection of SARS-CoV-2 with excessive inflammation as a result of the exaggerated immune response and elevated production of “immunocytokines” resulting in cytokine storm (CS); followed by a series of events, like acute organ damage, acute respiratory distress syndrome (ARDS) as well as death. Hence attempts to reduce cytokine storm are now being considered as a new paradigm shift in the clinical management of SARS-CoV-2. Tocilizumab (IL-6 blocker), Baricitinib (JAKs and AAK1 inhibitor), TNFα inhibitors (Infliximab, Adalimumab, Certolizumab) are currently being evaluated for possible block of the CS. Hence, rationalizing anti-inflammatory therapeutics would be the most judicious approach for significant reduction in COVID-19 mortality. In order to elucidate optimized and rationaled use of different therapeutics in COVID-19, we collated latest available information from emerging scientific evidences, integrated previous attempts as well as clinical successes, and various adopted approaches to mitigate past outbreaks with of SARS-CoV and MERS CoV.

## 1 Introduction

**Fig. 1:**
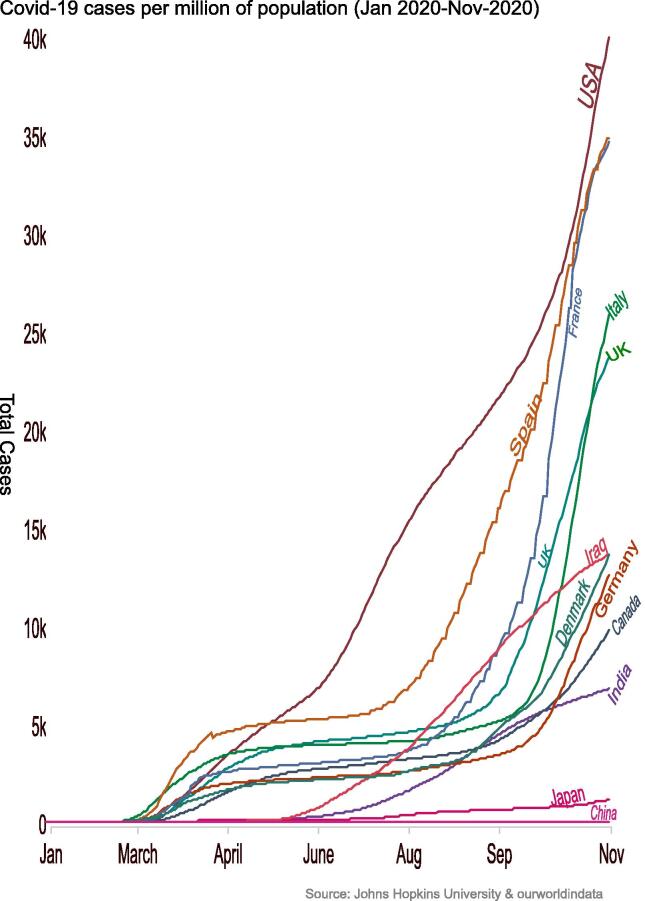
Covid-19 cases per million of population (Jan 2020-Nov-2020).

**Fig. 2:**
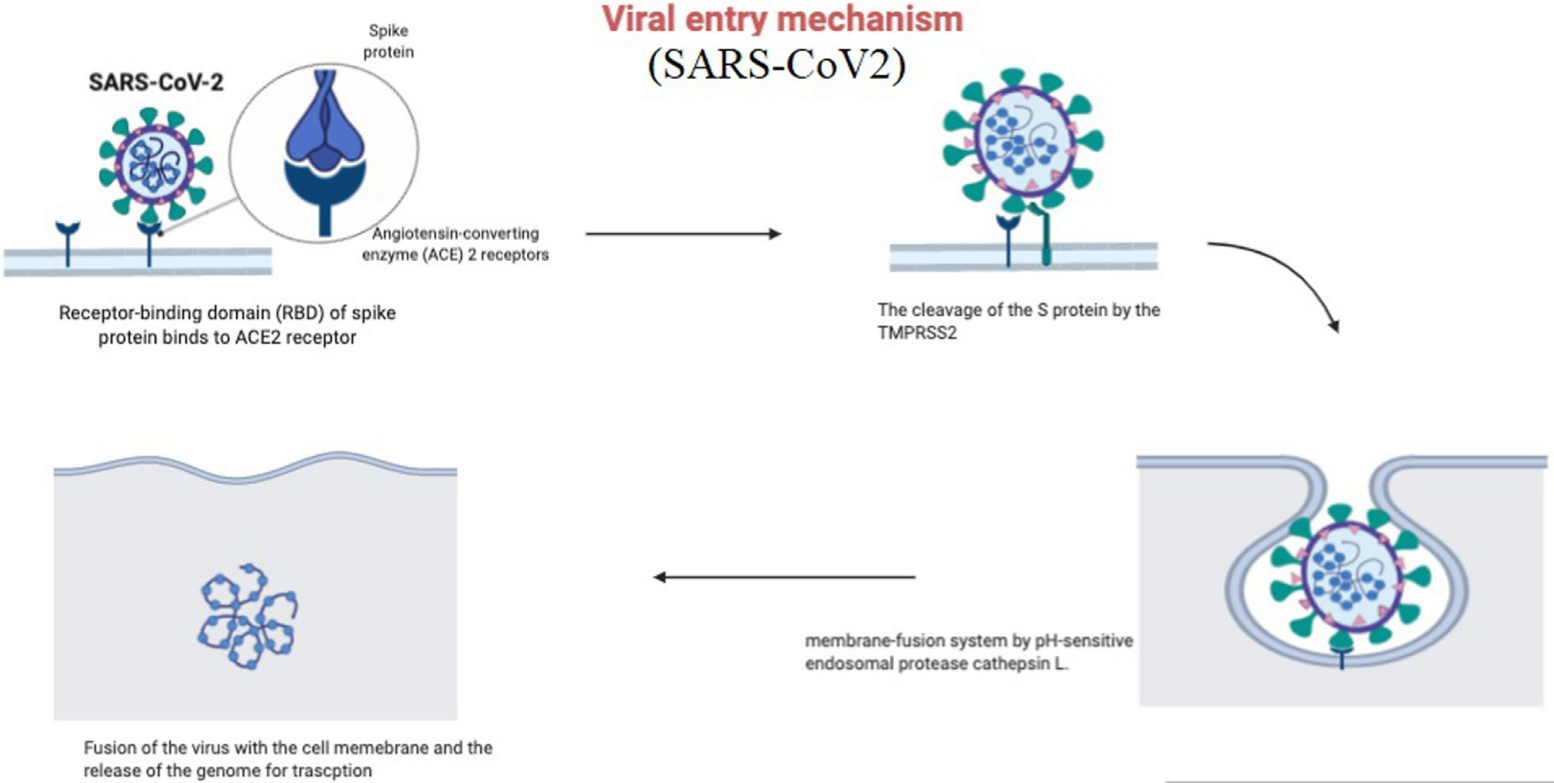
Entry mechanism of COVID-19.

SARS-C**oV-2** which possibly originated from Wuhan (China), was labeled as pandemic by the WHO. It is attributed with 64,845,925 reported cases from 213 countries and 1,499,357 deaths worldwide, till 3rd of Dec 2020 (“Coronavirus Update (Live),” n.d.). The SARS-C**oV-2** virus is still rapidly spreading, while total number of infected and dead patients are increasing worldwide **(**Fig. 1) (“COVID-19 deaths and cases,” n.d.; R. Zhang et al., 2020). Earlier infections with coronaviruses include: MERS-CoV- first identified in Saudi Arabia. While SARS coronavirus (SARS-CoV) was first reported in Guangdong province in China (“Middle East respiratory syndrome coronavirus (MERS-CoV),” n.d.; “WHO | SARS (Severe Acute Respiratory Syndrome),” n.d.). Coronaviruses are non-segmented and single stranded positive-sense RNA (+ssRNA) viruses with size around 30 kba, so known as the largest RNA virus. These single-stranded RNA viruses (+ssRNA) are abundantly present in many of isolated animal species, specifically bats, and rarely they cross species barriers and lead to outbreaks (“Vaccines for COVID-19,” 2020). SARS-CoV-2 exploits ACE2 receptors as entry points into human cells via S protein. After the endocytosis of S protein, the serine protease TMPRSS2 that is considered as an essential part in the entry process, cleaves the S protein at S1/S2 and the S2’ domain. Such cleavage enables the fusion of both viral as well as cellular membranes by the action of S2 subunit. Thus, TMPRSS2 inhibitors which block the entry of the virus are believed to slowdown the viral infections, and are now rationalized as promising therapeutics in clinical management of COVID-19 **(**Fig. 2**)** (Hoffmann et al., 2020). The symptoms such as fever, dry cough, myalgia, fatigue, and diarrhea, in severe cases progress to Acute lung injury/Acute respiratory distress syndrome (ALI/ARDS), failure of respiratory and heart functions, sepsis, and sudden cardiac arrest (N. Chen et al., 2020a, Huang et al., 2020, Rockx et al., 2020). However, pathological evidences from lung specimens of infected COVID-19 patients with severe ARDS, established significant damages; bilateral diffuse alveolar edema, pneumocyte desquamation as well as prominent hyaline membrane (R. Zhang et al., 2020). Interestingly in case of young generation who are diagnosed with COVID-19, the disease course is reported to be as silent, but their viral loads can infect others. Although some antibody testing methods are being rationalized but still RT-PCR is the standard test for diagnosis of coronaviruses. The attempts are also made towards CRISPR based technology as an alternative, cheaper and quicker diagnostic test (Metsky et al., 2020). To date, as of 11^th^ Jan 2020, there are no specific therapeutics or vaccines approved by WHO that have been designated in the clinical management of COVID-19, except restricted use of some novel vaccines for validation purposes only. Currently available treatments are supportive care, which reduce symptoms and mechanical ventilation which represents the main supportive intervention for patients with severe infection (Cascella et al., 2020). Hence, such compelling situations create a panic and indispensable event for urgent need of treatment modalities against COVID-19. Different drug design strategies are already being employed, which include approaches to explore prophylactics, vaccines, drug repurposing as well as novel molecules, the objective of this review is to highlight past outbreaks related to SARS-CoV as well as MERS-CoV type of viral infections and to envisage immune system-host responses to viral infections, in order to anticipate some better COVID-19 strategies and promising solutions. The integration of such vital and dynamic information with current strategies bears good promise to conceptualized novel immunomodulatory drugs, as both prophylactic and therapeutic vaccines, against COVID-19, with preferable reduction in post-viral“ immunocytokines” progression.

## 2 Structure of coronaviruses

**Fig. 3:**
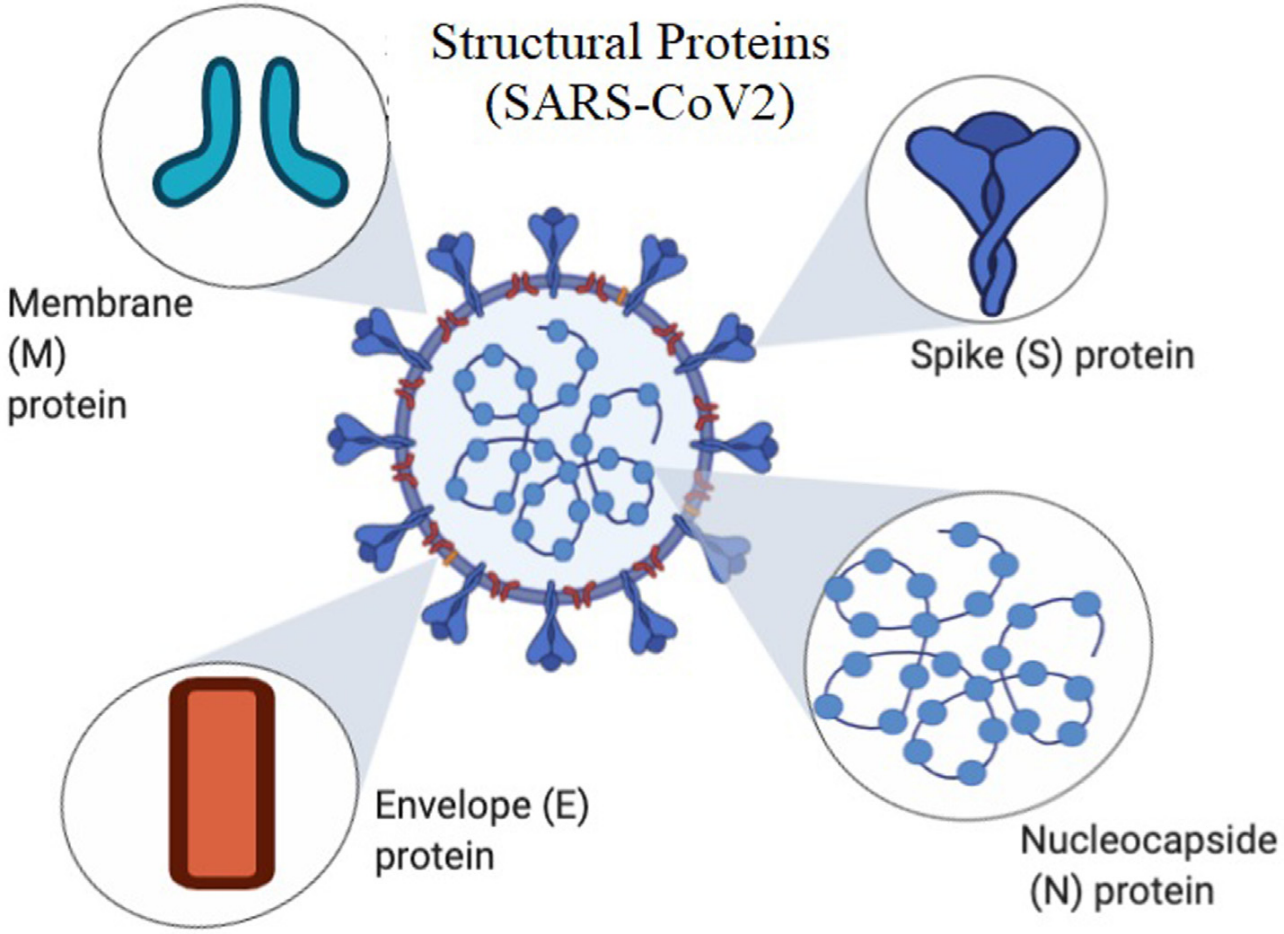
Structure of COVID-19.

Coronaviruses are collectively members of Coronaviridae family and subfamily of Coronavirinae; that are composed of 4 different genera: α-, β-, γ-and Δ- variants of coronavirus (Wu and McGoogan, 2020). CoV-virion is composed of 4 structural proteins. Spike (S) glycoprotein  are characteristically present on the surface of CoV viruses, and serve the essential purpose of establishing interaction and attachment with host cells. The M protein which is a type III transmembrane glycoprotein, composed of 3 different domains, imparts shape to the virus, it also facilitates the curvature of the membrane, and is responsible for the binding to the nucleocapsid. The envelope E protein that is located on the S protein is considered a crucial part for the viral pathogenesis and serves an important function in assembly and release of virions. The proteinious nucleocapsid N is usually composed of 2 different domains, which are able to bind RNA, it binds to non-structural protein 3 (nsp3), a protein to link the genetic material of the virus to the RTC, and helps in the packaging of the genome into the virions **(**Fig. 3**).** It shows that the N protein has an antagonistic effect for the interferon and viral encoded repressor of RNA interference, hence would favor replication of viruses (Y. Chen et al., 2020; G. Li et al., 2020). The virus uses the RNA for the translation of polyprotein 1a/1ab (pp1a/pp1ab). It uses protease and papain-like protease to process the polyprotein to form the nonstructural proteins (nsps). These nsps are essential for the formation of replication-transcription complex (RTC), located in double-membrane vesicles (DMVs) (Snijder et al., 2006). Subsequent to synthesis of subgenomic RNA (sgRNA) and the mRNA, transcription terminates followed by formation of newly formed leader RNA, as transcription regulatory sequences at the site located between each open reading frames (ORFs). While, sgRNAs as templates are used to synthesize various subgenomic mRNAs (Sawicki et al., 2007, Snijder et al., 2006). The typical coronavirus contains 10 open-reading frames ORFs (ORF1a/b), which alone represents 2/3rd of the gene. This ORF encodes 16 different nsps (Masters, 2006). Also it contains other accessory proteins that differ between each type of coronaviruses like: 3a/b protein, and 4a/b protein, HE protein (Hussain et al., 2005). Coronaviruses possess similarities in their genomic structure, around 58% on the nsp coding region and 43% similarities on the region that encodes the structural protein, and overall 54% at the whole genome level. This suggests that the structural proteins are more diverse between the coronaviruses in order to adapt for the new host (Kandeel et al., n.d.).

**Fig. 4:**
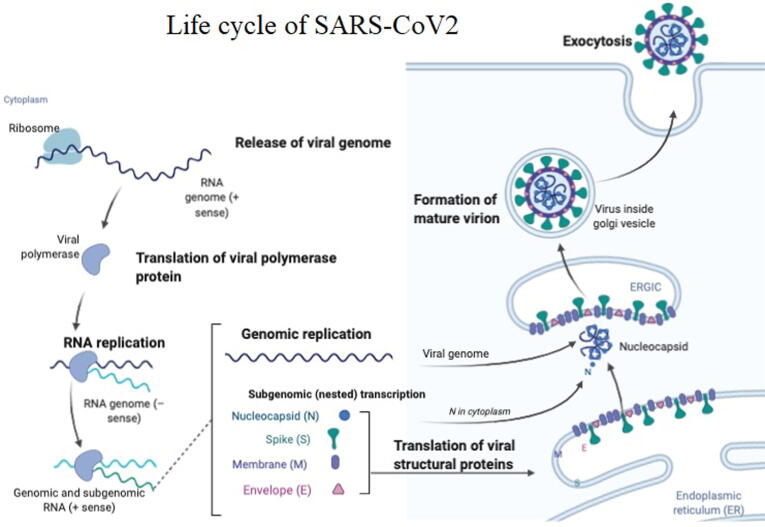
Life cycle of COVID-19 inside human cell.

The pathogenesis of the SARS-COV-2 is not completely understood but our knowledge with SARS-COV, MERS-COV pathogenesis gives an insight for the pathogenesis of SARS-COV-2 (Braciale et al., 2012). The attachment of S-protein to the angiotensin-converting enzyme 2 (ACE2) receptors over host cell surface forms virus-ACE2 complex, to be translocated within the endosome of the cells (Inoue et al., 2007). Inside the endosome, an endosomal acid proteases (cathepsin L) cleaves the S protein which will be fused into cell membrane complex (Simmons et al., 2005). Subsequent to this, viral genome is released, followed by their translation into viral replicase polyproteins pp1a and 1ab. These proteins further undergo cleavage via viral proteinases to mediate the synthesis of smaller viral proteins. The discontinuous transcription on the plus-strand genome forms the subgenomic negative-strand templates; to produce mRNAs. Then the, virus nucleocapsid is assembled from the genomic RNA and the N protein, sourced from cytoplasm. After which, these particles are transported to endoplasmic reticulum-golgi intermediate compartment (ERGIC). While, final step is the fusion of these elements with cell membrane to enable the release of virions (Stertz et al., 2007) **(**Fig. 4**)**.

## 3 Immune system and infection with viruses

**Fig. 5:**
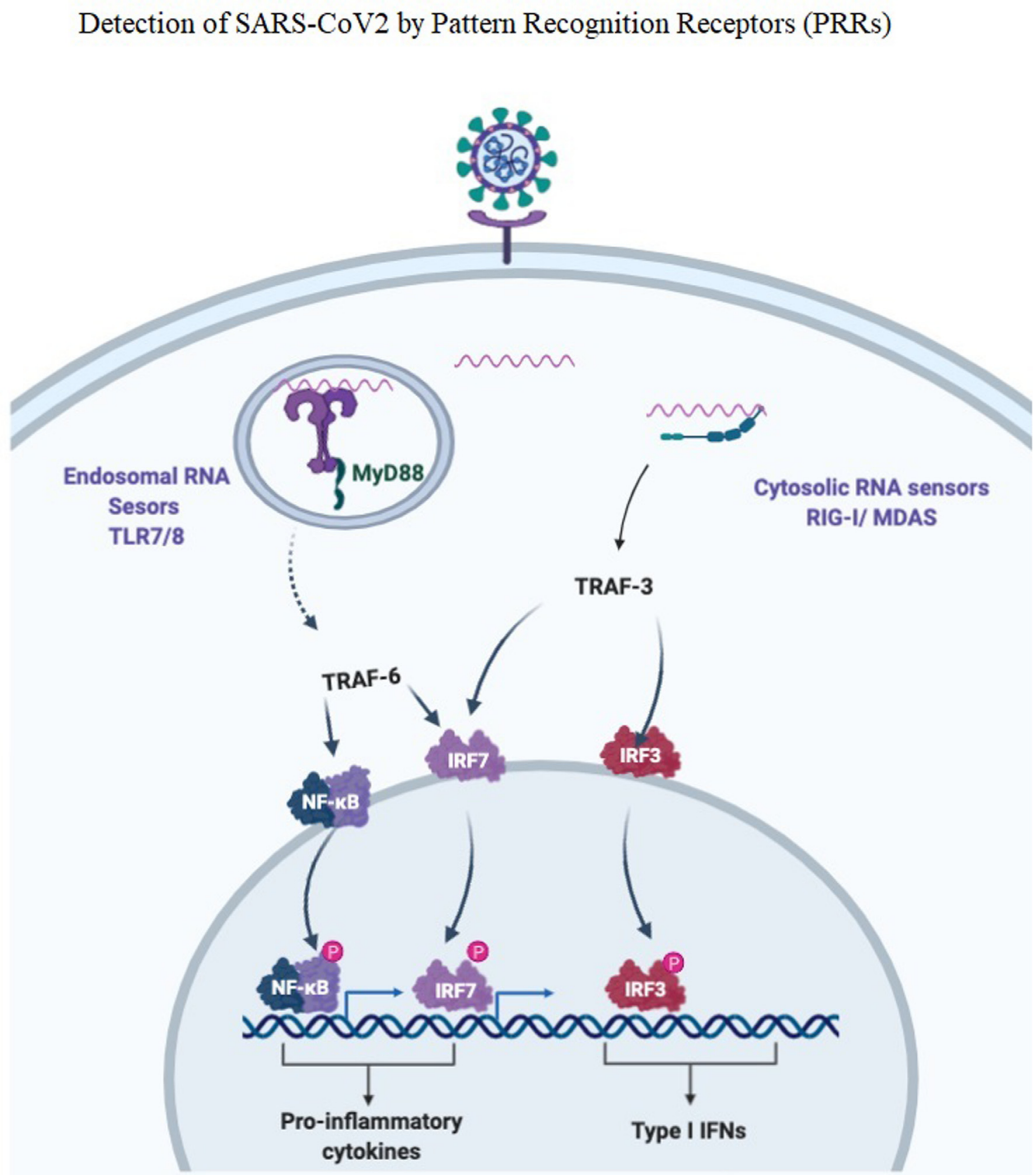
Detection of COVID-19 by pattern recognition receptors (PRRs).

The innate immune system may sense the presence of foreign microorganisms as well as their infection, through several types of pattern recognition receptors (PRRs) **(**Fig. 5**).** These receptors are capable of distinguishing pathogen-associated molecular patterns (PAMPs) like: viral tropisms, genomic structure of the virus (ssRNA, dsDNA, DNA). The PRRs are divided into transmembrane receptors and cytosolic receptors (G. Li et al., 2020). The transmembrane receptors like toll like receptors (TLRs) are able to recognize extracellular viruses, they are activated even without having active infection. TLRs are divided into cell surface TLRs which consist of (TLR 1,2,4,5,6) and endosomal TLRs which consist of (TLR 3,7,8,9). The TLR2, TLR4 can recognize viral proteins which results in the formation of IFN-β (Finberg et al., 2007, Xagorari and Chlichlia, 2008). On the other hand, the endosomal TLR can recognize viral genomic material, TLR3 can detect virus-derived dsRNA, TLR7/8 can detect virus derived ssRNA, while TLR9 recognizes virus-derived DNA. The signaling of TLR3 is mediated by TRIF molecule which results in the production of IFN-β, while other TLRs the MyD88 molecule mediate the production of IFN-α (Carty and Bowie, 2010). The endosomal TLRs are expressed on the surface of specific dendritic cells, for example TLR3 is expressed on CD8α(+) dendritic cells, while TLR7/9 is expressed on plasmacytoid dendritic cells. Generally, the innate immune system can detect viruses through limited and certain types of cells that expresse the transmembrane immune receptors (Blasius and Beutler, 2010).

Cytosolic receptors are expressed in all host cells. There are several types of cytosolic receptors which are able to detect viral genetic material, in the cytoplasm of the infected cells. RIG-I like receptors (RLRs) as well as NOD-like receptors (NLRs) are capable of recognising viral RNA, while ALRs can detect viral DNA(G. Li et al., 2020). RLRs consist of retinoic acid inducible gene-I (RIG-I) as well as melanoma differentiation associated gene 5 (MDA5); both of these genes are able to sense RNA of viruses inside the cytoplasmic region. Their signal is mediated by a single adaptor molecule, IPS-1 resulting in NF-κB production and the activation of IRF3/IRF7 (Schlee et al., 2009). The NLRs contain large different proteins that consist of a conserved NOD motif. The activation of NLRs will result in the formation of inflammasome resulting in the secretion of caspase-1-mediated IL-1β and IL-18 leading to INF-I production. The NLRP3 inflammasome is well known inflammasome that is activated by the presence of bacterial toxins, LPS, and viral RNAs (Ichinohe et al., 2009, Kanneganti, 2010).

The dsRNA that is formed by the virus replication process is high in CpG motifs (Schmitz et al., 2007). In contrast ssRNA is present in abundant amount in all host cells, the mechanism is not fully understood how the immune system will discriminate between viral and host RNA. One proposed mechanism is that the methylation status of the RNA, certain sequences of RNA, or restricted endosomal expression of TLRs (Hornung et al., 2006). While, activation of innate immune system is crucial because of favoring activation of adaptive immune response (G. Li et al., 2020). Recent studies showed that different pathways are activated according to the source of antigen, viral ssRNA will elicit the response of TLR7/MyD88 pathway, while CpG-DNA will elicit the response of TBK1 pathway (Stetson and Medzhitov, 2006). The adaptive immune response includes T cells activation as well as production of specific antibody (Ishii and Akira, 2007).

While, dendritic cells (DCs) which are being considered as important innate immune cells to establish the connection between the innate and adaptive immune responses (Klarquist et al., 2016). It is the strongest antigen-presenting cells that is capable of activating T lymphocytes and B-lymphocytes. Maturation of DCs is induced by GM-CSF, IL4, and TNFα, mature DCs can effectively activate T cells which can start, maintain, and regulate the immune response (Klarquist et al., 2016, Nouri-Shirazi and Guinet, 2012). The acquisition of antigen and the activation of immature DCs will result in their mobilization and migration to the lymph nodes that drains the infected tissue. Inside the lymph nodes, the DCs initiate the process of the adaptive immune responses to fight the virus (Braciale et al., 2012). Owing to antiviral functions, T cells are individually designated as CD4 + T cells, and CD8 + T cells. Briefly, the main function of CD4 + T cells is to trigger the process for virus-specific antibodies production via T-dependent B cells activation. While, cytotoxic CD8 + T cells kill viral infected cells (Cecere et al., 2012). After the migration of antigen presenting cells into the lymph nodes, the process of projecting viruses antigens through MHC class I molecules to CD8^+^ T cells takes place (Khanna et al., 2008). Subsequent to this, T cells reacts with MHC class I, to be induced and activated, followed by proliferation and differentiation into effector T cells, which migrate into infection site to start eliciting antiviral immune responses (Braciale et al., 2012). In order to maintain the production of formation and secretion of the cytokines by CD8^+^ T cells, it needs to bind to the co-stimulatory ligands on the APC which will provide signal strength that is important for the secretion of the pro-inflammatory cytokines by the T cells, which would induce B lymphocytes to be activated and differentiated (Hufford et al., 2011).

## 4 Immune system response to SARS-CoV-2/ COVID-19

### 4.1 Cell-mediated immune response

At the start of coronavirus pandemic, researchers lacked the understanding of how natural immunity develops to combat SARS-CoV-2. A few months later, studies have shown that infected individuals can produce antibodies that neutralize the virus, as well as T-cells that can recognize and kill the SARS-CoV-2 infected cells (“Why decoding the immune response to COVID matters for vaccines,” 2020).

Just over a 100 years ago the world had faced a serious flu pandemic (1918). Again for about a year, the world is being surrounded with a quite similar situation. However, with advancements in medicine, it has been easier to characterize and identify the contagious virus and subgroup it under the coronavirus family (Shah et al., 2020). Rapid and advanced genome sequence technique has helped in identifying the structure, function of the virus as well as its immunogenicity in different world populations. It also helped to adopt the proper prevention measures. The main target of the coronavirus is the lungs, causing pneumonia and lymphopenia in the infected individuals. Some of the components of the virus like spike and nucleocapsid proteins activate the immune response in the host to deplete the virus. These viral antigens can be either recognized by B cells or presented by MHC complexes with T-cells, resulting in antibody production, which leads to an increase in the secretion of cytokines, and cytolytic activity in the acute infection phase. MHC genetic polymorphism enables it to present some of the T cell epitopes very well over the other MHC alleles. MHC alleles associate and its downregulated expression has been linked to the severity of the disease against coronavirus and influenza (Shah et al., 2020). Studies have shown that recovered individuals induce strong protective responses by producing a memory T-cell pool against SARS-CoV and MERS-CoV. The memory cells will not persist for a long term, and when reactivated cause local damage because of cross-reactivity. The reports have suggested that the highly infectious SARS-CoV-2, shows related symptoms in there different stages and develop an exhaustive T-cell pool at higher viral loads (Shah et al., 2020). As there is no established vaccine or medicine for Covid-19, the body immune system remains the best defense. As long as the natural immune system functions in the normal way, covid-19 infections may go un-noticed. There are three types of immunity; innate immunity (rapid response), adaptive immunity (slow response), and passive immunity (Chowdhury et al., 2020).

As the number of COVID-19 patients continues to rise, identifying and understanding the immune response to SARS-CoV-2 infection becomes more essential to understating disease pathogenesis and developing vaccines with a long-term immune response. In COVID-19 patients, the strongest immune system responses to SARs-CoV-2 infection was directly related to the detected level of T-cell (mainly CD8 + and CD4 + ) due to their responses to the SARs-CoV-2 spike protein (S). The following inflammatory cells including lymphocytes, T-helper cells (Th), B cells, and natural killer/ cytotoxic T lymphocyte (CTL) and cytokines (mainly IFNγ, IL-2, and TNFα) have been also reported to be produced as a response to SARS-CoV-2 infection (Gregory A Poland et al., 2020b, Weiskopf et al., 2020). The T-cell responses to spike protein have been characterized and well-correlated with the magnitude of the IgG and IgA antibody titers. SARS-CoV-2 T-cells were also detected in low levels in 20% in some samples taken from healthy people who have not been previously exposed to SARS-CoV-2. This was justified due to infection with common cold coronaviruses and considered as an indication of indicative of cross-reactivity between common cold coronaviruses infection and SARs-CoV-2 Infection (Grifoni et al., 2020, Weiskopf et al., 2020). The CD8 + and CD4 + T inflammatory cells, which play a vital role in the clearance of viruses, were detected in 70% and 100% of the COVID-19 patients, respectively (Grifoni et al., 2020). The inflammatory status of COVID-19 was significantly associated with detected levels of lymphocytes, CD8 + and CD4 + T cells, B cells, and natural killer cells, particularly CD8 + T cells and the CD4+/CD8 + ratio (F. Wang et al., 2020). In both mild cases and severe cases, a decrease in the absolute numbers of T lymphocytes, CD4 + T cells, and CD8 + T cells were detected, but was more accentuated in severe cases (F. Wang et al., 2020). In severe cases, expression of IFN-γ by CD4 + T cells also tends to be lower than in moderate cases (G. Chen et al., 2020).

**Fig. 6:**
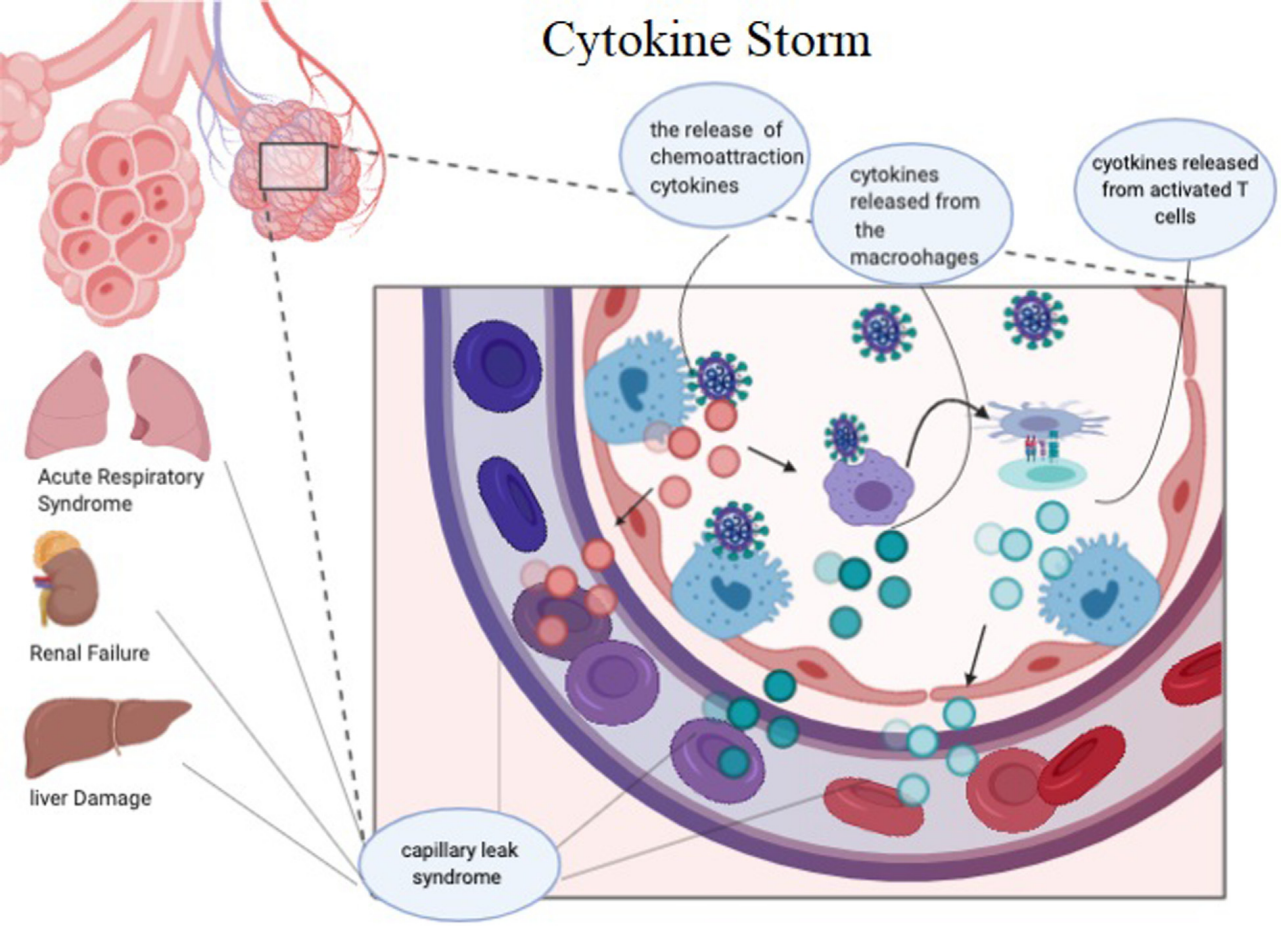
Cytokine storm.

The immune response to SARS-CoV2 occurs in two phases, the first phase is the incubation phase in which the adaptive immune response is essential to fight the infection and eliminate the effect of the virus in order to prevent the progression into the severe second phase. The progression to the next phase depends on the health status of the person and the HLA haplotype (Xu et al., 2020). If the person fails to stop the virus he will enter into the severe phase where strong inflammatory response which is damaging to the lungs and manifests as acute respiratory distress syndrome (ARDS) (Xu et al., 2020). ARDS is caused by cytokine storm events, which is indeed a systematic inflammatory response to favor burst release of cytokines (IFN-α, IFN-γ, IL-1β, IL-6, IL-12, IL-18, IL-33, TNF-α, TGFβ, etc.) and chemokines (CCL2, CCL3, CCL5, CXCL8, CXCL9, CXCL10, etc.) from the cells with immunological functions (X. Li et al., 2020, p. 19). The cytokine storm will trigger the damage specifically in tissues that express high number of ACE2, like: intestine and kidney (X. Li et al., 2020, p. 19; Xu et al., 2020). The treatment by the use of the Cytokine activated mesenchymal stem cells is what could stop the inflammatory process and enhance tissue repair (Wang et al., 2014) **(**Fig. 6**).**

The N structural protein of SARS-CoV has an antagonistic effect against the immune escape protein and the interferon response. In SARS-CoV-infected patients CD8 + T cells represent the majority of infiltrative inflammatory cells in the pulmonary interstitium which can clear the virus and induce cell injury. While the depletion of CD4 + T cell was linked to decreased number of lymphocytes in the infection site and the ability to neutralizing antibody and secretion and production of the cytokine, resulted in the delayed clearance of the virus from lungs and prolongation of viral replication (Maloir et al., 2018). SARS-CoV can mediate T cell apoptosis by binding BH3-like regions in c-terminal cyctolic domain of virus protein, with Bcl-xL. The depletion of T cell will result in the progression of the infection and promote virus survival (Yang et al., 2005). Several researches showed that the response of the T cells to the S structure protein and the other proteins such as M protein and N protein is persistent and will last for long time giving the prove for the synthesis of vaccine that is made of viral structural proteins (Braciale et al., 2012). The humoral immunity response that is mediated by B cells is important to fight the persistent phase of CoV infection. New researches have shown that the antigen stimulation of MERS-CoV is located in the S glycoprotein at the position 437 to 445 as evident by the epitope9-mer peptide “CYSSLILDY”(Ababneh et al., 2019). The computerized simulation has shown that this sequence has the greater B cell antigen formation and the highest interactions with MHCI alleles (Ali et al., 2014).

A very new research on November 2020 which was carried out by Shane Crotty at the La Jolla Institute for Immunology in California and his colleagues. They analyzed markers of the immune response in blood samples from 185 people who had a range of COVID-19 symptoms; 41 study participants were followed for at least 6 months (Dan et al., 2020). They found out that participants’ immune responses varied widely. But several components of immune memory of SARS-CoV-2 tended to persist for at least 6 months. Among the persistent immune defenders were memory B cells, which jump-start antibody production when a pathogen is re-encountered, and two important classes of T cell: memory CD4 + and memory CD8 + T cells. The results have not yet been peer-reviewed.

Another new research on November 2020 had reported that a widespread variant of the new coronavirus is able to evade the immune response that some people acquire after the infection is subsided (Thomson et al., 2020). Since the commencement of the pandemic, scientists have identified thousands of viral mutations in the genomes of SARS-CoV-2 samples taken from infected people. Gyorgy Snell at Vir Biotechnology in San Francisco, California, David Robertson at the University of Glasgow, UK, and their colleagues have examined a N439K mutation in a protein that the virus uses to invade cells (Thomson et al., 2020).

### 4.2 Antibody-mediated immune response and protective immunity

The antibodies produced by the infected body or through vaccination against SARS-CoV-2 are considered very crucial for the neutralisation and clearance of the virus. The antibodies are measured by using in-vitro assays and considered as good markers for detecting the effectiveness of the natural immune responses towards SARS-CoV-2 infection (Gregory A Poland et al., 2020). The antibodies formation responses to SARS-CoV-2 infections starts to show up between day 10 and day 22 after the exposure to the virus. This includes virus-specific IgM, IgA, and IgG indicating that antibodies mediate protective immunity to SARS-CoV-2 (Ni et al., 2020, Padoan et al., 2020).

Scientific research reported that the overall antibody response against SARS-CoV-2 in many ways resembles those for SARS-CoV-1, especially, the level of IgG and IgM after 7–14 days (Lou et al., 2020). In another study which focused on the assessment of the kinetics of the antibodies in the population produced in response to COVID-19 infection. It was reported that IgA antibodies were produced at the first week of infection and maximized in concentration at 20–22 days. IgM antibodies reached high levels at 10–12 days, then subsequently waned 18 days after the symptoms have been started (Ni et al., 2020; Gregory A Poland et al., 2020). A research which included 67 COVID-19 patients after symptom onset has found that the levels of IgM and IgG antibodies were significantly higher in patients with severe disease status than in those with milder disease status. IgM (up to 1:800) and IgG (up to 1:60), and were linked to the clinical outcomes (Tan et al., 2020, p. 19). This suggests that strong antibody responses may be an indication of more severe disease, while low antibody responses may be an indication of higher rates of clearance of the virus. In mild cases of the disease, detection of the antibodies (i.e., IgM, IgG) may take longer (four weeks or more) and in a few number of cases antibodies may not be detected at all at least at the time scale of the study. IgM and IgG antibodies to SARS-CoV-2 develop between 6 and 15 days after the onset of the disease (Wölfel et al., 2020, Zhao et al., 2020b). According to the recently available data for total antibodies, IgM and then IgG, the median seroconversion time was day-11, day-12 and day-14 post the appearance of the symptoms, respectively. Antibodies were detected in less than 40% of patients within 1 week of onset and surged to 100% (total antibodies), 94.3% (IgM) and 79.8% (IgG) from day-15 after onset (Zhao et al., 2020a).

The longevity of the antibody response is still unknown, but other coronaviruses have shown to produce antibodies that decrease over time (range: 12 to 52 weeks after symptom onset) and infections have been shown to recur (Kellam and Barclay, 2020). SARS-CoV-2 antibody levels of IgM and IgG may be maintained for seven weeks (Xiao et al., 2020) or at least 80 percent of cases until day 49 (Zeng et al., 2020). In comparison, IgG antibodies for SARS-CoV-1 are maintained longer than those of SARS-CoV-2 (2 and 3 years in 90% and 50% of patients infected with SARS-CoV-1, respectively) (Wu et al., n.d.). Furthermore, the detection of IgA antibodies in the nasal cavity may be important as serum IgA antibodies were not elevated. IgA persisted in the nasal mucosa for one year after infection with seasonal coronavirus 229E (Callow et al., 1990).

## 5 Immunomodulators in COVID-19 infection

The current SARS-CoV-2 related findings in regard to genetics, pathophysiology and immune responses established closed linkage with SARS and MERS. While, in patients with severe COVID-19, aggressive and unregulated pro-inflammatory cytokines release i.e., Cytokine storm (CS) are common attributes. Hence, lymphopenia and “cytokine storm” are being considered as critical factors in COVID-19 pathogenesis.(W. Zhang et al., 2020). While, the process of cytokine storm leads to lethal acute lung injury (ALI) and acute respiratory distress syndrome (ARDS), it is emerging as major therapeutic target for the development of novel vaccines and as prevention tools, followed by treatment (Channappanavar and Perlman, 2017; R. Zhang et al., 2020). Hence among the various treatment modalities, several attempts were also taken to interfere with the process of cytokine storm, methods to block the CS as well as to explore optimal stage where such anti-inflammatory therapy would be most efficient against the infections. This review targets immune response of COVID-19 infection by judicious integration of scientific information from previous SARS-CoV and MERS-CoV outbreaks. These insights would enable the design of appropriate vaccines and drugs of immunomodulatory activity; to control post-viral infections, “immunocytokines” (W. Zhang et al., 2020) **(Table −1).**

### 5.1 Interlukin-6 (IL-6) blockers

SARS-CoV-2 stimulates the generation and secretion of cytokines molecules (cytokines storm) such as IL-2, IL-6, IL-7, GSCF, IP10, MCP1, MIP1A, and TNFα. While, the elevated level of proinflammatory cytokines in severe cases also resembles CS in SARS-CoV-1 and MERS (Russell et al., 2020).In another cohort study, post-treatment corona + ve survived patients exhibited a relatively higher level of IL-6. A similar observation was also made to link critical stage of the disease with elevated level of IL-6 during COVID-19. Therefore, the cytokines storm is blamed to be the main cause of the lung tissue damage, which proceeds to oedema, air-exchange dysfunction, Acute Respiratory Distress Syndrome, acute cardiac injury, including deaths. Subsequently, elevated levels of IL-6 were associated with the severity of pulmonary complications (Russell et al., 2020). Therefore, IL-6 has been suggested as a potential immuno-therapeutic target for the treatment of SARS-CoV-2 infection and some other similar viral infections as MERS and SARS (Russell et al., 2020). One class of these drugs, which serve as interleukin-6 inhibitors or IL-6 inhibitors- Tocilizumab (TCZ), is a recombinant human IL-6 monoclonal antibody which blocks IL-6 signaling to inhibit inflammatory response. While, apart from conventional use of Tocilizumab in rheumatic diseases, such as rheumatoid arthritis; owing to its potent anti-IL6R effects, Tocilizumab was also approved by US FDA in CAR-T cell cytokine release syndrome (“Coronavirus COVID-19 (SARS-CoV-2) | Johns Hopkins ABX Guide,” n.d.). Several other studies, where TCZ was used on COVID-19 patients, with other antiviral agents revealed that the symptoms of the treated patients improved significantly and 75.% had improved oxygenation (Fu et al., 2020; C. Zhang et al., 2020). In addition, the percentage of peripheral lymphocytes was found to return back to normal in more than half of the treated patients (W. Zhang et al., 2020). Owing to its promising therapeutic significance (response) in the above mentioned data, scope of TCZ within the treatment of severe COVID-19 cases is being rationalized. While, outcome of several reports, which also involve severe COVID-19 related pneumonia cases; namely clinical trials established both safety and efficacy profile of Tocilizumab, as a monotherapy (ChiCTR2000029765) as well as part of pharmaceutical combination (ChiCTR2000030442 and ChiCTR2000030894)(W. Zhang et al., 2020).

### 5.2 Janus kinase (JAK) inhibitors

In immune system, central cellular responses towards exogenous signals are being mediated by JAK-STAT signaling pathway whereas enzyme Janus kinases (JAKs) play regulatory functions owing to their interaction with signal transducer as well as activator of transcription proteins (STATs). Hence, inhibition of JAK-STAT serves the purpose of drug target in inflammatory disease such as cancer and rheumatoid arthritis. **(**Li et al., 2013, Russell et al., 2020**)**.This indicates that JAK-STAT pathway is a promising therapeutic target to prevent entry of coronaviruses well as inflammatory mediated complications.

Virus’s replication process, including SARS-CoV-2, starts by entry of virus into host cells through receptor-mediated endocytosis. This endocytosis process is mediated by binding of the viral spike protein with the cell-surface receptors (Phadke and Saunik, 2020). Several reports confirmed that SARS-CoV-2 S-spike protein binds with the cell-surface ACE2 receptors in the AT2 alveolar epithelial cells (Cai et al., 2020). After being bound to the cell-surface ACE receptors, the endocytosis process starts which is regulated by Janus kinases (JAKs) and AP2-associated protein kinase enzymes (AAK) such as the well-known AP2-associated protein kinase 1 (AAK1). Therefore, for the purpose of slowing viral entry/endocytosis into the lung cells as well as reduction in infectious viral spreading, the exploration of AAK1 blockers is emerging as another promising approach (Russell et al., 2020; W. Zhang et al., 2020).

Baricitinib is a relative safer and tolerable JAKs and AAK1 inhibitor being approved in February 2017 by EMA (European Medical Agency) for clinical management of moderate-to-severe active rheumatoid arthritis among those who are previously showing insufficient response with standard anti-rheumatic based drug therapy (Liu et al., 2020, Phadke and Saunik, 2020, Richardson et al., 2020). Hence, Baricitinib, due to its favourable affinity profile, may reduce viral-entry into the host cell, so is considered as a potential candidate to slowdown COVID-19 infection (W. Zhang et al., 2020). Subsequently, due the safety as well as the efficacy of JAK inhibitors against COVID-19 cases, many drugs such Jakotinib, Ruxolitinib are also being explored in clinical trials (ChiCTR2000030170) (Russell et al., 2020). In this regard, many JAK Inhibitors and anti-IL6 agents due to their inhibitory function against inflammatory cytokines such as INF-α; are still being investigated as important drug targets to reduce viral response (Price et al., 2020).

### 5.3 Tumour necrosis Factor-α (TNFα) inhibitors

Tumour necrosis factor (TNF) is a family of proteins, receptors and cytokines, which acts as cell signaling involved in systemic inflammation. So they are considered potential targets for drugs such as TNFα inhibitors, which are used as anti-inflammatory suppressants in immune-mediated disorders including, rheumatoid, psoriasis and inflammatory bowel disease. A research study also established that TNFα mediated immune pulmonary injuries with coronavirus (SARS) infections. Hence, some monoclonal antibodies such as Infliximab, Adalimumab, Certolizumab which selectively inhibit TNFα can be purposed in COVID-19 infections (Tobinick, 2004a). Subsequently, despite the wide use of such inhibitors in several clinical conditions, still no any adverse impacts or threats with the use of TNFα inhibitors are yet documented, namely in COVID-19 patients (Russell et al., 2020, Waljee et al., 2020).

### 5.4 Viral entry inhibitors (via dendritic Cells) (DCs)

Among the immune cells of mammalian, dendritic cells (DCs) process antigen materials followed by ensuring presence of antigen on the cell surface of T cells. Hence these cells are also often being designated as antigen-presenting cells. Such unique characteristic enables them to serve role as messenger in between innate and adaptive immune systems. As described above regarding the viral entry to the host cells, literature reported that SARS-CoV can invade and enter host-cells through receptor-mediated endocytosis. This entry into host cells is mediated by the transmembrane spike (S) glycoprotein with cell-surface receptors which was confirmed to be ACE2 in the case of SARS-CoV-2 (Cai et al., 2020, Walls et al., 2020). A recent study suggested that ACE2 is not the only cell-surface receptors that SARS-CoV-2 can utilize to bind to- and enter the host cell. This suggestion was made based on findings regarding the SARS-CoV, where ACE2, DC-SIGN and L-SIGN cell-surface receptors were found to be utilized for binding followed by entry within host cells (Cai et al., 2020). While, such SARS-CoV-2 binding with ACE2 receptors followed by entry are being favored by monocytes and DCs transfer through DC-SIGN(Chen and Subbarao, 2007, Yen et al., 2006). In the same report they suggested that smoking causes up-regulation of the expression of the ACE2 and DC-SIGN in the AT2 and DC lung cells, respectively, and hence could enhance the virus entry (Cai et al., 2020). To conclude, DC-SIGN antagonists are expected to block the DC-SIGN receptors and thus disrupt the viral entry into host-cells which is mediated by interaction between the viral S-protein with the DC-SIGN receptor, specifically, in the smoking patients.

### 5.5 Antigen based vaccines: The Ligand antigen epitope Presentation system (LEAPS):

In several infectious diseases where causative antigenic peptide(s) sequences are already revealed, Ligand Antigen Epitope Presentation System serve purpose as emerging platform technology to design antigen-specific immunotherapeuticals against such diseases. Therefore, these antigens have been specifically designed to stimulate the desired immune responses but without eliciting any of clinically significant undesired inflammatory response related to damage of lung tissues. Hence such platform technology can be used to produce antiviral and anti-inflammatory immunotherapeutical peptides. However due to its specificity, these immunotherapeuticals are specifically designated against those viruses, of which sequence was exploited as directing elements. In lieu of the above facts, in various immuno-compromised infectious disorders, such as tumors, auto-immune diseases, allergy as well as SARS-CoV-2 type of viral diseases, these immunotherapeuticals would serve best purpose as an ideal protective vaccine. The main proposed advantage of this LEAPS technology is to design immunotherapeuticals using specified antigen sequence which are less variable between different strains of the same causative virus followed by relatively lesser probability of showing any mutation in response to antibodies being produced by prior infection or other exposed vaccines candidates. Hence antigen sequence of many of glycoprotein spike in infectious viral strains, are being explored as important and promising elements to be exploited in production of such vaccines. For example, CEL-SCI Corporation (NYSE American: CVM) revealed its LEAPS peptide technology would utilize conserved regions of coronavirus proteins as promise solution hence by stimulating protective cell mediated T cell responses as well as reduction in viral load would protect against SARS-CoV-2 coronavirus (Martin, n.d.).

## 6 Current status of vaccines for SARS-CoV-2

In order to mitigate the recent SARS-CoV 2 pandemic more than 120 vaccines had been investigated through different phases of preclinical and clinical studies. Different approaches had been adopted to develop vaccines including viral proteins, inactivated virus, live attenuated virus, peptide based and nucleic acid based vaccines (Gregory A. Poland et al., 2020). The FDA had issued several guidelines for the requirements to secure the license for COVID-19 vaccine such as: the data from the preclinical phase, the characteristics of the immune response that occurs on animal model, the toxic effects of the vaccine and the minimum efficacy of the vaccine (Schwartz, 2020). The data generated from the preclinical studies indicated the following: to neutralize antibodies, the S protein is considered as major target (Coleman et al., 2014), most of the antibodies are targeting the RBD portion of the S protein (Du et al., 2013), vaccine generated antibody neutralization are considered protective (Munster et al., 2017). Trialsdone on MERS vaccine showed that DNA based vaccine of the S protein can elicit a robust antibody response, many vaccines have been able to induce cellular immunity that is enough to reduce viral load (Haagmans et al., 2016). The current status of vaccines are; 01 vaccine has been approved in china to be used by their military, 03 vaccines are under investigation in phase 3, 08 vaccines are still in phase 2, 11 vaccines are in phase-1, and the remaining are in the preclinical studies (Gregory A. Poland et al., 2020). Live virus or inactivated virus vaccines are considered the most effective type of vaccine that can induce immunity but might have several safety issues (Stauft et al., 2019). In India, Codagenix Inc and the Serum Institute of India are using this approach to develop the vaccine, they are using the codon-deoptimization technique to produce attenuated virus. This technique is shown to be safe also it can produce immunologic response when used in influenza (Mueller et al., 2020). The University of Hong-Kong are in the process of developing an intranasal live attenuated vaccine that expresses the S protein (Gregory A. Poland et al., 2020). Subunits vaccines are selected proteins from the virus or in some cases fragments of the proteins that are able to produce an immune response (Gregory A. Poland et al., 2020). In this approach there is no active infection thus allaying the safety concerns. Most of the vaccines in this approach are focusing on the S protein or specific domains of the S protein like the RBD (N. Wang et al., 2020). There are some vaccines that target the N protein based on previous studies conducted on SARS CoV-1 and MERS (which reported the production of antibodies), and it contains HLA -restricted T-cell epitopes (Liu et al., 2006). Usually the selected proteins are combined with adjuvant to enhance the immune reaction. The production of large scale antigen could be problematic (Shanmugaraj et al., 2020). Baylor college of medicine are investigating the effect of recombinant proteins of SARS CoV-1 in the protection against SARS COV-2. Novavax are using protein nanoparticle vaccine, they are using as adjuvant a saponin-based Matrix‐M adjuvant which has been used in Ebola and MVA vaccines and showed to produce a good adaptive immune response (Bengtsson et al., 2016, Magnusson et al., 2018; Gregory A. Poland et al., 2020). University of Queensland are developing a protein based vaccine, while using the molecular clamp technique to stabilize the virus protein into a correct 3 dimension shape, which will allow the immune system to develop humoral immunity against the conformational epitope. Vaxart are trying to develop a tablet vaccine using nonreplicating adenovirus as vector for the delivery of S protein (recombinant) with TLR adjuvant into the epithelium of the mucosa. The mayo clinic vaccine research group is developing a vaccine which is peptide based derived from natural SARS CoV-2 proteins (processed from several viruses which have been identified via mass spectrometry) (Ovsyannikova et al., 2007). Virus like particles (VLP) vaccines are virus shell without the nucleic acid therefore considered noninfectious vaccines, while remaining in the 3 dimensional structure and its antigenic property can illicit an immune response (Mohsen et al., 2017). Multiple groups are working with this approach to develop vaccine such as the University of Pittsburgh produced a skin patch vaccine that neutralized antibody production in animal models (Kim et al., 2020). Nucleic acid vaccines using RNA and DNA are rapid and inexpensive approach that do not use live viruses. DNA vaccine has several drawbacks; it needs sophisticated delivery system, also it is difficult to produce large doses of the vaccine. On the other hand RNA vaccines might exhibit other issues like tranfection efficiency in vivo (Gregory A. Poland et al., 2020). Several groups are using this approach to develop the vaccine; Sanofi and the Biomedical Advanced Research and Development Authority, The Karolinska Institute and Cobra Biologics are working on DNA vaccine (Pang et al., 2020). While The NIH’s Rocky Mountain Laboratories, Tongji University in China, The Imperial College London all of them are developing RNA vaccines (Gregory A. Poland et al., 2020). The vaccine that has been developed in the USA by National Institute of Health (NIH) and Moderna is mRNA 1237 vaccine which is type of non-replicating RNA vaccine that would stimulate S protein production in the host cells, inturn producing antibodies against it (Jackson et al., 2020). Pfizer Inc and BioNTech are investigating 04 different mRNA vaccines, 02 of them are nucleoside modified mRNAS, one is mRNA that contain uridine, and the other is self amplifying RNA. The promising result of the clinical trials on the BNT162b1 vaccine showed an excellent immune response with mild adverse symptoms such as headache, pain at the injection site, and fatigue (Gregory A. Poland et al., 2020).

Vector based vaccines are live attenuated viruses that consist of safe vector such as adenovirus, MVA that expresses coronavirus proteins for immunization, usually can not replicate inside human cells, but some vectors can have some limited replication capabilities. The advantage of this approach is that the safety profile and immunogenicity has been already established for the other pathogens, which will accelerate the vaccine development (Gregory A. Poland et al., 2020). CanSino Biologics Inc is using adenovirus type 5 vectored vaccine, currently in the phase 2 of clinical trials. The NIH’s Rocky Mountain Laboratories in collaboration with the University of Oxford are evaluating chimpanzee adenovirus vectored vaccine. Pasteur Institute, Themis Bioscience GmbH, and the University of Pittsburgh Center for Vaccine Research are studying vaccine that used measles virus as a vector with the expression of the S protein.

### 6.1 Sinovac vaccine (CoronaVac)

The randomized, double blinded, placebo controlled trials were conducted on inactivated vaccine from sinovac, involving 600 healthy individuals aged 18 - 59 years. They used 02 different doses, 03 and 06 Âµg, alumunium hydroxide was used as the adjuvant. The doses were given 2–4 weeks apart. Results were evaluated using ELISA for the immunogenicity and cytopathogenic effect for the antibody neutralization. There were no major side effects from the 02 doses. The 4 week dose showed better neutralization results in comparison to the 02 weeks. Around 90% of the participants had seroconversion. According to age, the titer was better in the age group between 18 and 39 (Y.-J. Zhang et al., 2020).

### 6.2 Sinopharm inactivated whole virus vaccine

The phase I and phase II clinical trials of this vaccine involved, 96 subjects for phase I, and 224 for phase II studies, within the age group 19–59 years. Phase I trial used 3 different doses with 4 weeks duration between the 2 doses, the doses were: 2.5,5,10 μg. In phase 2 the dose was 5 μg with 2 or 3 weeks interval. The safety of the vaccine was compared to coronaVac vaccine, the neutralization titer ranged from 1:121 to 1:316. Currently, the vaccine is in phase III trials (Xia et al., 2020).

### 6.3 CanSino vaccine AdV5 vaccine

This vaccine exploits the adenovirus type 5 as a vector which expresses the S protein. The vaccine is licensed to be used in Chinese military. The phase II trials of the vaccine involved 508 individuals with the doses of 5x10^9^ and 1x10^11^ virus particles. Immunity and antibody neutralization to the RBD were evaluated after 28 days of vaccine administration. Antibody neutralization result were 59% seroconversion for the high dose, 47% seroconversion for the small dose. The result considered low, it might be due to pre-existing immunity to adenovirus type 5. The T cell response in the beginning of the trial was under the level of the detection but increased after the vaccine administration to 90% response for the higher dose and 88% for the lower dose. In terms of side effects, the vaccine appeared to be reactogenic notably with the high dose. Symptoms such as fever, headache, and fatigue were common, pain at the injection site occurred in more than half of the participants. Fever which is considered as grade 3 adverse reaction occurred, 9% in high dose group and 1% in the low dose group. Currently the vaccine in the phase III with the lower dose 5x10^5^virus particles (Zhu et al., 2020).

### 6.4 AstraZeneca vaccine (ChAdOx1 nCoV-19)

This vaccine is collaboration between Astrazaneca, Oxford university, and Serum institute of India. ChAd01 vector is being used, which is non replicating vector with the expression of wild type, full length S protein. The published results for the phase I and II clinical trials, indicated that 1,077 individuals received a dose of 5x10^10^viral particle once, except for 10 participants who recieved another dose at day 28. The placebo group received meningitis vaccine for safety comparison. Immune response essays were done using authentic SARS CoV-2. Interferon gamma linked immunospot assay was used to determine the cellular immune response. The result showed excellent seropositivity titer that reached in median to 100%. The T cell immunity response peaked at day 14 reaching 856 SFU per 10^6^ which got reduced to 424 SFU at day 65. For the side effects of the vaccine 70% experienced fatigue, 60% experienced headache. Other common symptoms that occurred were increased temperature. Compared to the meningitis group the ChAdOx1 nCoV-19 had lower safety profile. The vaccine is under investigation in several countries now for single vs double dose vaccine (Folegatti et al., 2020).

### 6.5 Moderna mRNA-1273 vaccine

The phase I study of this vaccine involved 45 participants aged 18–55 years, the participants received 2 doses, 28 days apart, three doses were tested : 25, 100, 250 μg. After the first dose the higher dose showed higher antibody response as evident by ELISA test. After the second dose their was evidence of antibody neutralization that was confirmed by 2 methods. The side effects reported in around 50% of the participants were muscle pain, pain at the injection site, headache, chilis, and fatigue. The systemic manifestation was reported after the second, particularly with the higher dose (Jackson et al., 2020).

### 6.6 Pfizer BNT162b1 and BNT162b2 vaccine

Pfizer and BioNTech are developing, **BNT162b1**, which is a mRNA vaccine that is delivered in LNP, and encodes the RBD of the spike protein of SARS-CoV-2. In phase I/II trials 45 subjects were vaccinated at doses 10, 30, 100 μg. It is given in double doses with 3 weeks between them. Neutralization titer and RBD binding IgG titer were high and increased with the dose and after the second dose. The safety profile of the vaccine showed the symptoms were mild to moderate and they were dose dependent. The second high dose of the 100 μg was not given because of increased reaction and because of increased immunity after the second 30 μg dose (Mulligan et al., 2020)**. BNT162b2** is similar to the previous one but it encodes the full length S protein. The antibody titer in both vaccines were comparable. But the safety profile was better in the BNT162b2 group. Also it was tested on old age groups (65–85 years). The company decided to go with BNT162b2 for phase III (Walsh et al., 2020).

### 6.7 Novavax NVX-CoV2373 vaccine

This vaccine is recombinant full length S protein. The formulation contains a saponin-containing adjuvant. The clinical trail recruited 131 participants, 02 doses were evaluated 5,25 μg, the period between the prime and the second dose was 3 weeks, there was group who did not received the second dose. The outcome was tested using ELISA and microneutralization technique using authentic SARS CoV 2 and CD4 cytokine staining stimulated with S peptide. There was intermediate response after the first dose for both doses (5,25), after the boost dose seroconversion reach 100%. The results suggest that there is no difference in the response between the high and the small dose. The T cell response was robust in both doses as evident by the CD4 response. Headache, malaise and fatigue were the most common side effect. Fever was rare in occurrence. The vaccine is now under phase II and III trials (Keech et al., 2020).

## 7 Conclusion

There are no specific drugs for treatment of infection with SARS-CoV-2 that are approved by FDA. However, there are some FDA approved treatments which might help in reducing the symptoms of the disease. Chloroquine and Hydroxychloroquine are antimalarial drugs that are believed to interfere in viral infection by elevating endosomal pH, in addition to interfering with the glycosylation of ACE2 receptor. While adenosine triphosphate analogues such as Remdesivir, owing to mimicking structure of adenosine triphosphate, serves role of incorporation competitor against adenosine triphosphate unit. Hence instead of standard requirement of adenosine triphosphate in viral RNA-dependent RNA polymerase (RdRp) process of Ebola virus, incorporation of Remdesivir would consequence into delayed RNA chain terminator functions. So the above outcome would prospect towards rationalized purposing of Remdesivir in viruses with similar attributes. However some studies have failed to show the efficacy of these drug in COVID-19 states, it is thereby imperative to discuss the viral mediated chronic inflammatory events which are more prominent in latter phase of viral infections and remain unabated and leading cause of ARDS-mediated death. The, efficiency of many existing drugs is limited in terms of efficient actions during initial phase of viral infection, rather than latter phase of disease progression. Such outcomes create a limiting factor and barrier on clinical prospect and translation of these drugs, in late phase of SARS-CoV-2. Subsequently, post coronavirus infection, NF-κB activation is mainly mediated by MyD88 pathway via pattern recognition receptors (PRRs), to induce different pro-inflammatory cytokines, such as IL-6, TNFα and chemokines production. Thus designing appropriate drugs with immunomodulatory characteristic would serve prophylactic/therapeutic vaccines in the treatment of COVID-19 infection, by reducing impact of “immunocytokines” on viral infectivity and disease progression.

## Declaration of Competing Interest

The authors declare that they have no known competing financial interests or personal relationships that could have appeared to influence the work reported in this paper.
